# Prehospital hypothermia is associated with increased mortality

**DOI:** 10.1186/2197-425X-3-S1-A374

**Published:** 2015-10-01

**Authors:** J Shaw, B Taylor, K Thies

**Affiliations:** Army Medical Directorate, British Army, Camberley, United Kingdom; Cardiothoracic Anaesthesia, University Hospital of South Manchester, Manchester, United Kingdom; British Army, Stafford, United Kingdom; University Hospital of Birmingham, Birmingham, United Kingdom

## Introduction

Trauma is leading cause of death among people under the age of 40.[1] Accidental hypothermia, acidosis, and coagulopathy represent the lethal triad in severely injured patients. [2] There is good evidence that unintended hypothermia worsens outcomes after major trauma.[3] We aimed to audit the frequency of hypothermia among seriously injured patients presenting to a UK major trauma centre.

## Objectives

We aimed to examine if trauma patients had a temperature recorded within an hour of arrival in the emergency department. We examined if hypothermia on hospital admission was associated with a difference in mortality, ICU or hospital length of stay, or discharge to residential care for the first time.

## Methods

All patients presenting to the Emergency Department of a single UK major trauma centre over a six-month period (October 2013 to April 2014), with major trauma (ISS ≥15) had a review of their case notes.

## Results

We identified 261 patients who had suffered major trauma during the audit period. 109 patients were excluded from analysis: 71 were secondary transfers from other hospitals, 24 had an extremely delayed presentation (>24 hours from time of injury), five patients had ISS < 15 on review and notes were unavailable for nine.

Of the remaining 152 patients, no temperature was recorded within the first hour for 80 patients - the most common reason documented was the presence of collar and blocks making access use of tympanic thermometers difficult. All patients had a temperature recorded eventually while in the emergency department.

Of patients found to be hypothermic on admission, 7/30 (23.3%) died prior to hospital discharge, whereas 2/42 (4.8%) of normothermic patients died prior to hospital discharge. This finding was statistically significant using a 2-tailed Fishers exact test (p = 0.0289).

There was no significant difference in length of hospital or ICU stay, or discharge to residential care in our sample.

## Conclusions

Hypothermia was relatively common among a sample of patients presenting to a UK major trauma centre with significant trauma. It was associated with an increased mortality, re-iterating the importance of hypothermia as part of the lethal triad following major trauma.

Our audit demonstrated that this important sign was frequently missed as temperature was not recorded within the first hour of hospital admission.
